# Clinical characteristics and prognostic value of pre‐retreatment plasma epstein‐barr virus DNA in locoregional recurrent nasopharyngeal carcinoma

**DOI:** 10.1002/cam4.2339

**Published:** 2019-07-03

**Authors:** Ming‐Zhu Liu, Shuo‐Gui Fang, Wei Huang, Han‐Yu Wang, Yun‐Ming Tian, Run‐Da Huang, Zhuang Sun, Chong Zhao, Tai‐Xiang Lu, Ying Huang, Fei Han

**Affiliations:** ^1^ Department of Radiation Oncology Sun Yat‐sen University Cancer Center Guangzhou Guangdong Province People's Republic of China; ^2^ State Key Laboratory of Oncology in South China Guangzhou Guangdong Province People's Republic of China; ^3^ Collaborative Innovation Center for Cancer Medicine Guangzhou Guangdong Province People's Republic of China; ^4^ Guangdong Key Laboratory of Nasopharyngeal Carcinoma Diagnosis and Therapy Guangzhou Guangdong Province People's Republic of China; ^5^ Department of Radiation Oncology Hui Zhou Municipal Centre Hospital Huizhou Guangdong Province People's Republic of China

**Keywords:** clinical characteristics, Epstein‐Barr Virus DNA, prognostic, recurrent nasopharynx

## Abstract

**Purpose:**

To define the clinical characteristics and prognostic value of pre‐retreatment plasma Epstein‐Barr virus (EBV) DNA, we investigated EBV status in locoregional recurrent nasopharyngeal carcinoma (lrNPC) patients.

**Methods:**

Between April 2008 and August 2016, the data of patients with nonmetastatic lrNPC were retrospectively reviewed. The survival indexes of patients between different pre‐retreatment EBV status groups were compared.

**Results:**

A total of 401 patients with nonmetastatic lrNPC were enrolled, and 197 (49.1%) patients had detectable pre‐retreatment plasma EBV DNA. Treatment included radiotherapy alone (n = 37 patients), surgery alone (n = 105), radiotherapy (n = 208), surgery combined with radiotherapy (n = 20), chemotherapy and targeted therapy (n = 31). Median follow‐up was 32 months. The 3‐year locoregional relapse‐free survival (LRRFS), distant metastasis‐free survival (DMFS), and overall survival (OS) rates for the entire cohort were 64.8%, 89.4%, and 58.8%, respectively. The estimated 3‐year LRRFS, DMFS, and OS rates for the pre EBV‐positive group vs the pre EBV‐negative group were 54.2% vs 75.0% (*P* < 0.001), 86.6% vs 91.9% (*P* = 0.05), 51.6% vs 65.9% (*P* = 0.01), respectively. Among patients in the clinical stage rI/II, there were 17 patients in the radiotherapy alone group and 49 patients in the surgery alone group. And there was no significant difference in overall survival between radiotherapy and surgery, even among the different pre‐EBV statuses (*P* > 0.05). In terms of long‐term toxic and side effects, the incidence of radioactive temporal lobe injury in the radiotherapy group was higher than that in the surgery group (35.3% vs 8.2%, *P* < 0.001), and no statistically significant difference was found in other long‐term toxic and side effects.

**Conclusions:**

The positive rate of pre‐retreatment plasma EBV DNA in lrNPC is lower than primary NPC. The prognosis of EBV DNA negative group is better than positive group. For locally early‐stage lrNPC, regardless of EBV DNA status, radiotherapy and surgery are available options and both can achieve better long‐term survival.

## 1 INTRODUCTION

Nasopharyngeal carcinoma (NPC) is prevalent in Southeast Asia. Globally, there were an estimated 86 700 new cases of NPC and 50 800 deaths in 2012.^1^ The endemic form of this disease is invariably associated with prior Epstein‐Barr virus (EBV) infection.^2^ Clinical outcomes have improved dramatically over the past three decades because of advances in disease management.^3^ Many studies^4^, ^5^ have demonstrated that EBV can be widely detected in the primary and metastatic cancer cells of almost every patient with NPC. Additionally, the plasma EBV DNA load has been demonstrated to be correlated with tumor burden, since plasma EBV DNA may derive from the cancer cells.^6^, ^7^, ^8^, ^9^, ^10^, ^11^

Up to now, high levels of pre‐ and posttreatment plasma EBV DNA load were associated with poor survival outcomes. Pretreatment plasma EBV DNA was detectable (>0 copy/mL) in 53.9‐93.0% of patients with NPC, and it was closely related to clinical stage and treatment outcomes.^12^, ^13^, ^14^, ^15^, ^16^, ^17^, ^18^, ^19^, ^20^, ^21^, ^22^ During posttreatment follow‑up, plasma EBV DNA has considered to be a good marker for predicting distant metastasis and overall survival (OS) but not locoregional recurrence in patients with NPC.^11^, ^21^

With improvements in radiation therapy techniques, diagnostic imaging and concurrent chemotherapy in loco‐regionally advanced disease, treatment outcomes and disease control rates have been improved substantially.^23^ However, local recurrence and distant metastasis remain as the main causes of treatment failure. Local recurrence still occurs in 5%‐10% of the NPC patients, even when treated with advanced techniques such as intensity modulated radiation therapy (IMRT).^24^, ^25^, ^26^, ^27^ Our previous study of long‐term outcomes and prognostic factors of re‐irradiation for locally recurrent NPC indicated that salvage re‐irradiation by IMRT improve local tumor control and prolong patient survival.^28^

In the practice, patients who suffered from recurrent NPC sometimes have consistently undetectable plasma EBV DNA when diagnosed with locoregional recurrent NPC (lrNPC) that develops second tumor recurrence or distant failure or death, whereas some patients with elevated EBV DNA levels remain disease‐free even after long‐term follow‐up. Moreover, a recent study by Weng et al^29^ retrospectively investigated sixty‐two patients with local residual or recurrent NPC treated with endoscopic nasopharyngectomy plus chemoradiotherapy (CRT) or with CRT alone, which concluded that pre‐retreatment serum EBV‐DNA level was associated with disease prognosis, and patients with local intermediate‐ and late‐stage rNPC, especially those negative for EBV‐DNA, may consider opting for surgery followed by postoperative adjuvant radiotherapy or chemotherapy.

However, there was no convincing large‐scale study to evaluate the characteristics of pre‐retreatment plasma EBV DNA level for patients diagnosed with lrNPC. Additionally, the evidence of the value of pre‐retreatment plasma EBV DNA in patients with lrNPC has not yet been described and demonstrated. Therefore, we performed this retrospective study to further investigate the clinical features and value of pre‐retreatment plasma EBV DNA for predicting prognosis in patients with lrNPC.

## 2 MATERIALS AND METHODS

### 2.1 Patient selection

We retrospectively analyzed the data from the patients diagnosed with locoregional recurrent nasopharyngeal carcinoma (lrNPC) who were treated between April 2008 and August 2016 at Sun Yat‐sen University Cancer Center (Guangzhou, China). All patients underwent conventional therapy before being diagnosed with lrNPC. The inclusion criteria were (a) being diagnosed by pathology or medical history and imaging of the sites of recurrence that were difficult to biopsy, clinically difficult to perform biopsy by minimally invasive means, such as the sinus cavernous, (b) having no evidence of distant metastases, (c) have a plasma EBV DNA assay when diagnosed with lrNPC. The exclusion criteria were (a) previous other malignant cancers, (b) absence of secondary malignancy, pregnancy or lactation, or (c) having accepted other treatment after diagnosis with lrNPC. The clinical research ethics committee of Sun Yat‐sen University Cancer Center approved this study.

### 2.2 Clinical staging

Routine staging included a complete medical history, physical examination, magnetic resonance imaging (MRI) of the skull base and entire neck, chest and abdominal computed tomography (CT), a whole‐body bone scan, and ^18^F‐fluorodeoxyglucose (18F‐FDG) PET/CT if indicated. All patients were restaged according to the 8th edition of the Union for International Cancer Control and American Joint Committee on Cancer (AJCC) staging system.^30^ Two radiologists separately evaluated scans to assure that all medical images had minimal heterogeneity in restaging, and disagreements were resolved by consensus.

### 2.3 Real‐time quantitative EBV DNA PCR

Measurement of plasma EBV DNA used real‐time quantitative PCR. Samples of peripheral blood (5 mL each) were collected and centrifuged at 1600 g for isolation of plasma. DNA from plasma samples was extracted with the QIAamp Blood Kit (Qiagen, Hilden, Germany) and measured using a real‐time quantitative polymerase chain reaction assay amplifying the BamHI‐W region of the EBV genome. The sequences of the forward and reverse primers were 5′‐GCCAG AGGTA AGTGG ACTTT‐3′ and 5′‐TACCA CCTCC TCTTC TTGCT‐3′, respectively.^31^, ^32^ In our institution, a plasma EBV DNA concentration of <0 copies/mL was defined as undetectable.

### 2.4 Clinical treatment

The treatment regimens of recruited patients included radiotherapy (RT), CRT, surgery (S), S + CRT, and chemotherapy (CT). The RT technology was IMRT, and the planning target volume (PTV) doses were 60‐70 Gy at 1.8‐2.3 Gy/fraction. Chemotherapy included adjuvant chemotherapy, concurrent chemotherapy, and neoadjuvant chemotherapy, which were mainly cisplatin‐based. Surgery mainly included nasopharyngectomy and selective cervical lymph node dissection. Various surgical approaches for nasopharyngectomy included open maxillary‐swing nasopharyngectomy, endoscopic resection or trans‐oral resection.

### 2.5 Follow‑up

Follow‐up was measured from the first day of therapy to the last examination or death. Patients were examined at least every three months during the first two years, with follow‐up examinations every six months thereafter until death. The routine follow‐up workup included physical examination, plasma EBV DNA assay, nasopharyngeal fiberoptic endoscopy, nasopharyngeal and neck MRI, chest X‐ray or computed tomography (CT), liver ultrasound or CT, whole‐body bone scan, and 18F‐FDG‐PET/CT if necessary. The end points (time to first defining event) were locoregional relapse‐free survival (LRRFS), distant metastasis‐free survival (DMFS), and OS, and OS was set as the primary endpoint.

### 2.6 Statistical analysis

Life‐table estimation was performed according to the method of Kaplan‐Meier. Univariate comparison of survival curves was performed with the use of log‐rank test. The multivariate Cox proportional hazards model was used to estimate hazard ratios (HR) and 95% confidence intervals (CI). All statistical tests were two‐sided, and differences with *P* < 0.05 were considered statistically significant.

Variables in this study included age, sex, Karnofsky Performance Status (KPS), presence of severe late complications, disease‐free interval, recurrent T stage, recurrent N stage, recurrent clinical stage, treatment regimen, and pre‐retreatment EBV status. The relation between the plasma EBV DNA concentration and the baseline data was evaluated with the use of a chi‐square test. All statistical tests were two‐sided, and a *p* value of less than 0.05 was considered to indicate statistical significance. Analyses were performed with the use of SPSS software (version 20.0, SPSS Inc, Chicago, IL, USA).

## 3 RESULTS

### 3.1 Patient characteristics

**Figure 1:**
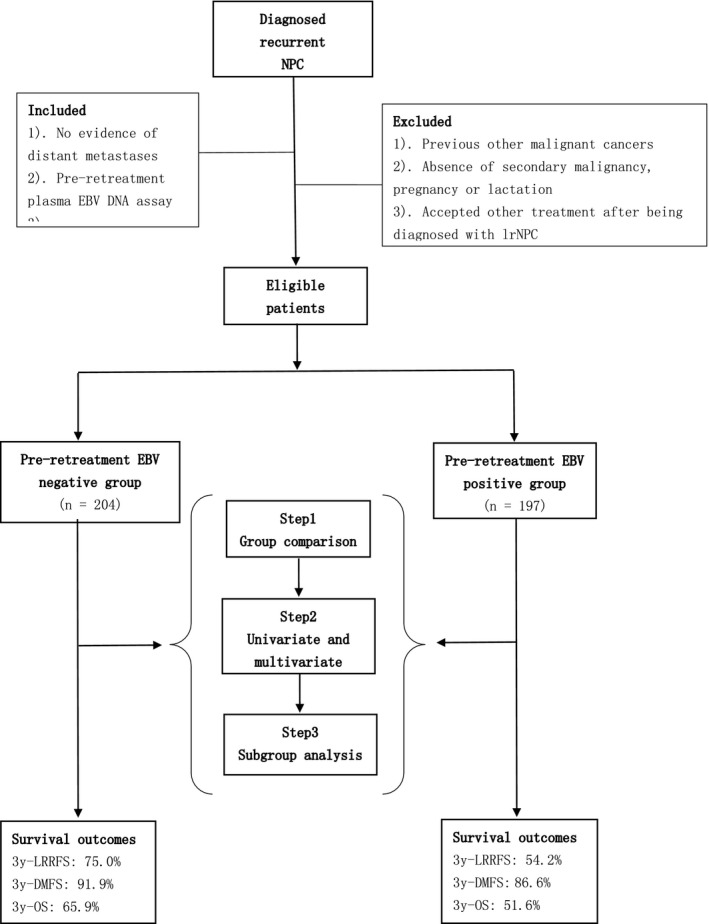
CONSORT flow diagram

A total of 401 patients treated between April 2008 and August 2016 were retrospectively enrolled. We conducted group comparisons, univariate and multivariate analysis and subgroup analyses based on all 401 eligible cases (Figure 1). The clinical characteristics are summarized in Table 1. In the entire cohort, 197 (49.1%) patients with detectable pre‐retreatment plasma EBV DNA when diagnosed with lrNPC were classified as the pre EBV‐positive group, and the remaining with undetectable were classified as the pre EBV‐negative group. The median follow‐up time for this entire cohort was 32 months (range 3‐118 months). Median age was 46 years (range 22‐76 years), and 308 (76.8%) patients were male. In terms of the host factors, gender, age, KPS, disease‐free interval, and presence of severe late complications were similar between the pre EBV‐positive group and pre EBV‐negative group (*P* > 0.05). However, more patients in the pre EBV‐positive group had tumor burdens classified as rT4 (*P* < 0.001), rN1‐3 (*P* = 0.004) and recurrent clinical stage IVA (*P* < 0.001) as compared with the pre EBV‐negative group.

**Table 1:** Clinical characteristics of 401 patients with locoregional recurrent nasopharyngeal carcinoma

Characteristic	Pre^a^ EBV‐negative No. (%)	Pre EBV‐positive No. (%)	*P* b
Gender			0.525
Male	154 (75.5)	154 (78.2)	
Female	50 (24.5)	43 (21.8)	
Age (years)			0.334
≤45	102 (50.0)	89 (45.2)	
>45	102 (50.0)	108 (54.8)	
KPS			0.980
≥90	152 (74.5)	147 (74.6)	
<90	52 (25.4)	50 (25.4)	
DFI (months)^c^			0.266
0‐12	36 (17.6)	29 (14.7)	
13‐24	64 (31.4)	49 (24.9)	
25‐36	37 (18.1)	38 (19.3)	
>36	67 (32.9)	81 (41.1)	
Presence of severe late complications			0.228
Yes	55 (27.0)	56 (28.4)	
No	149 (73.0)	141 (71.6)	
Recurrent T stage^d^			<0.001
rT0‐1	66 (32.4)	40 (20.3)	
rT2	25 (12.3)	17 (8.6)	
rT3	75 (36.7)	71 (36.1)	
rT4	38 (18.6)	69 (35.0)	
Recurrent N stage^d^			0.004
rN0	129 (63.2)	94 (47.7)	
rN1	63 (30.9)	76 (38.6)	
rN2	11 (5.4)	21 (10.6)	
rN3	1 (0.5)	6 (3.1)	
Recurrent clinical stage^d^			<0.001
rI	28 (13.7)	9 (4.6)	
rII	57 (28.0)	33 (16.8)	
rIII	81 (39.7)	82 (41.6)	
rIVA	38 (18.6)	73 (37.0)	
Treatment regimen			0.011
RT alone	25 (12.3)	12 (6.1)	
CRT	92 (45.1)	116 (58.9)	
S alone	64 (31.4)	41 (20.8)	
S + CRT	8 (3.9)	12 (6.1)	
CT alone	15 (7.3)	16 (8.1)	

Abbreviations: AJCC, American Joint Committee on Cancer; CT, chemotherapy; CRT, chemoradiotherapy; EBV, Epstein‐Barr virus; KPS, Karnofsky Performance Status; RT, radiotherapy; S, surgery; UICC, Union for International Cancer Control.

a pre, pre‐retreatment.

b *P* values were calculated using the chi‐square test or Fisher exact test if indicated.

c DFI, Disease‐free interval was defined from the date of completion of treatment to diagnosis of recurrence or final follow‐up if sooner.

d According to the 8th edition of the AJCC/UICC staging system.

### 3.2 Pre‐retreatment plasma EBV DNA level

The mean concentration of EBV DNA in plasma from the patients with nasopharyngeal carcinoma was 14416.9 copies/mL (range, 0‐640 000). The mean pretreatment plasma EBV DNA levels were described as stratified by different recurrent classifications. Advanced recurrent T category, recurrent N category, and recurrent clinical stage had higher mean pre‐retreatment plasma EBV DNA levels (*P* < 0.001; Table 2).

**Table 2:** Pre‐retreatment plasma EBV DNA level in patients with locoregional recurrent nasopharyngeal carcinoma of different recurrent classifications

Recurrent classification	No.	Mean pre‐retreatment plasma EBV DNA (copies/ml)	*P* a
Recurrent T category b			<0.001
rT0‐1	106	4905.6	
rT2	42	11206.9	
rT3	146	13574.8	
rT4	107	26248.3	
Recurrent N category^b^			<0.001
rN0	223	6396.4	
rN1	139	14300.7	
rN2	32	36469.7	
rN3	7	171422.9	
Recurrent clinical stage^b^			<0.001
rⅠ	39	535.9	
rⅡ	89	4745.2	
rⅢ	162	12188.9	
rⅣA	111	30300.4	

Abbreviations: AJCC, American Joint Committee on Cancer; UICC, Union for International Cancer Control.

a *P* values were calculated using the chi‐square test or Fisher exact test if indicated.

b According to the 8th edition of the AJCC/UICC staging system.

### 3.3 Treatment outcomes and failure patterns

The outcomes of failure patterns and mean pre‐retreatment plasma EBV DNA levels of different disease status are shown in Table 3. The patient who developed distant metastatic had higher mean pre‐retreatment plasma EBV DNA than the patient who only developed second locoregional failure. By the last follow‐up, 82 (41.6%) patients in the pre EBV‐positive group and 50 (24.5%) patients in the pre EBV‐negative group suffered from second locoregional failure, and 24 (12.2%) patients in the pre EBV‐positive group and 14 (6.9%) patients in the pre EBV‐negative group developed distant metastatic failure. There was a significant difference observed between the two groups in second locoregional failure (*P* = 0.01). However, there was no significant difference observed between the two groups in distant metastatic failure (*P* = 0.098). The pre EBV‐positive group had a higher percentage of cancer‐related death (*P* = 0.035).

**Table 3:** Pre‐retreatment plasma EBV DNA of disease status in pre EBV‐negative and pre EBV‐positive patients with locoregional recurrent NPC

Disease status	Mean pre^a^ plasma EBV DNA (copies/ml)	Pre EBV‐negative No. (%)	Pre EBV positive No. (%)	*P* b
Failure patterns				
Locoregional only	9379.9	41 (38.7)	65 (32.9)	0.025
Distant only	25244.5	5 (2.5)	7 (3.6)	0.530
Locoregional + distant	64496.2	9 (4.4)	17 (8.6)	0.108
Total locoregional	20236.1	50 (24.5)	82 (41.6)	0.010
Total distant	52100.9	14 (6.9)	24 (12.2)	0.098
Total	20653.5	55 (27.0)	89 (45.2)	0.009
Death				0.035
Yes and due to cancer	21321.5	104 (51.0)	121 (61.4)	
No	5590.0	100 (49.0)	76 (38.6)	

a pre, pre‐retreatment.

b *P* values were calculated using the chi‐square test or Fisher exact test if indicated.

### 3.4 Univariate analysis and multivariate analysis

**Figure 2:**
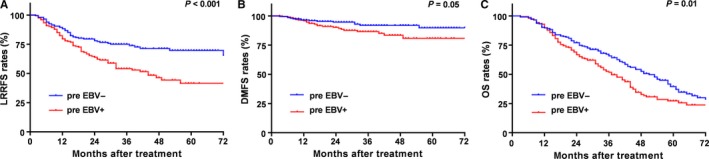
Kaplan‐Meier LRRFS. (A) DMFS (B) and OS (C) curves for locoregional recurrent NPC patients classified into pre‐retreatment EBV‐negative and pre‐retreatment EBV‐positive groups. LRRFS, local‐regional relapse‐free survival; DMFS, distant metastasis‐free survival; OS, overall survival

From the univariate analysis, we found that pre EBV‐positive status was correlated with poorer clinical outcomes significantly. The 3‐year LRRFS, DMFS, and OS rates for the entire cohort were 64.8%, 89.4%, and 58.8%, respectively. The estimated 3‐year LRRFS, DMFS and OS rates for pre EBV‐positive group vs the pre EBV‐negative group were 54.2% vs 75.0% (*P* < 0.001), 86.6% vs 91.9% (*P* = 0.05), 51.6% vs 65.9% (*P* = 0.01), respectively, (Figure 2). The 3‐year OS rates for staged rI, rII, rIII, and rIVA were 93.5%, 77.3%, 56.4%, and 58.8%, respectively.

In multivariate analysis including gender, age, KPS, presence of severe late complications, disease‐free interval, recurrent T category, recurrent N category, recurrent clinical stage, treatment regimen and pre EBV status as covariates, pre EBV status was an independent prognostic factor for LRRFS (HR, 1.95; 95% CI, (1.36‐2.79); *P* < 0.001). The pre EBV‐positive group had worse DMFS and OS than the pre EBV‐negative group. However, pre EBV status was not an independent prognostic factor for DMFS. Moreover, recurrent N category was an independent prognostic factor for DMFS. Gender, recurrent T category, and presence of severe late complications were independently associated with an increased risk of death. The findings from univariate and multivariate analyses are summarized in Table 4.

**Table 4:** Summary of univariate and multivariate analysis of 401 Patients with locoregional recurrent NPC

Endpoint	Variable	Univariate analysis		Multivariate analysis	
		HR (95% CI)	*P* b	HR (95% CI)	*P* b
LRRFS					
	Pre^a^ EBV status	1.94 (1.37‐2.77)	<0.01	1.95 (1.36‐2.79)	<0.001
DMFS					
	Recurrent N stage	2.66 (1.22‐5.81)	0.01	2.27 (1.02‐5.02)	0.044
	Pre EBV status	1.93 (0.99‐3.74)	0.05	1.89 (0.96‐3.73)	0.067
OS					
	Gender	0.61 (0.43‐0.86)	<0.01	0.70 (0.50‐0.99)	0.045
	Presence of severe late complications	1.47 (1.10‐1.96)	<0.01	1.37 (1.02‐1.85)	0.036
	Recurrent T stage	3.16 (2.26‐4.43)	<0.01	2.16 (1.44‐3.24)	<0.001
	Pre EBV status	1.40 (1.07‐1.82)	0.01	1.20 (0.92‐1.57)	0.179

Abbreviations: CI, confidence interval; DMFS, distant metastases‐free survival; EBV, Epstein‐Barr virus; HR, hazard ratio; LRRFS, loco‐regional relapse‐free survival; NPC, nasopharyngeal carcinoma; OS, overall survival.

The following variables were included in the Cox proportional hazards model with backward elimination: gender (male vs female), age (>45 y vs ≤45 y), KPS (≥90 vs <90), presence of severe late complications (yes vs no), disease‐free interval (0‐12 vs 13‐24 vs 25‐36 vs >36), recurrent T stage (T0‐1 vs T2 vs T3 vs T4), recurrent N stage (N0 vs N1 vs N2 vs N3), recurrent clinical stage (I vs II vs III vs IV), treatment regimen (RT vs CRT vs S vs S + CRT vs CT), pre‐retreatment EBV status (negative vs positive).

a pre, pre‐retreatment.

b *P* values were calculated using an adjusted Cox proportional hazards model.

### 3.5 Subgroup analysis of treatment regimen stratified by pre EBV status

**Figure 3:**
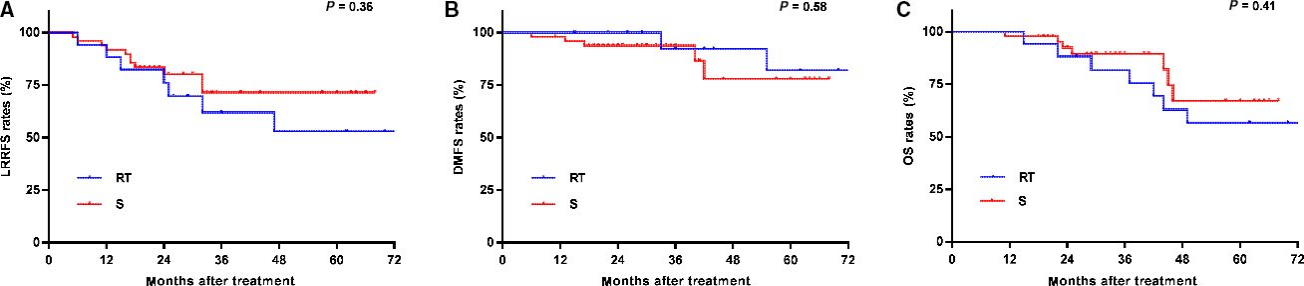
Kaplan‐Meier LRRFS. (A) DMFS (B) and OS (C) curves for locoregional recurrent NPC patients with rI+rII stage and stratified by treatment with RT alone and S alone. LRRFS, local‐regional relapse‐free survival; DMFS, distant metastasis‐free survival; OS, overall survival; RT, radiotherapy; S, surgery

**Figure 4:**
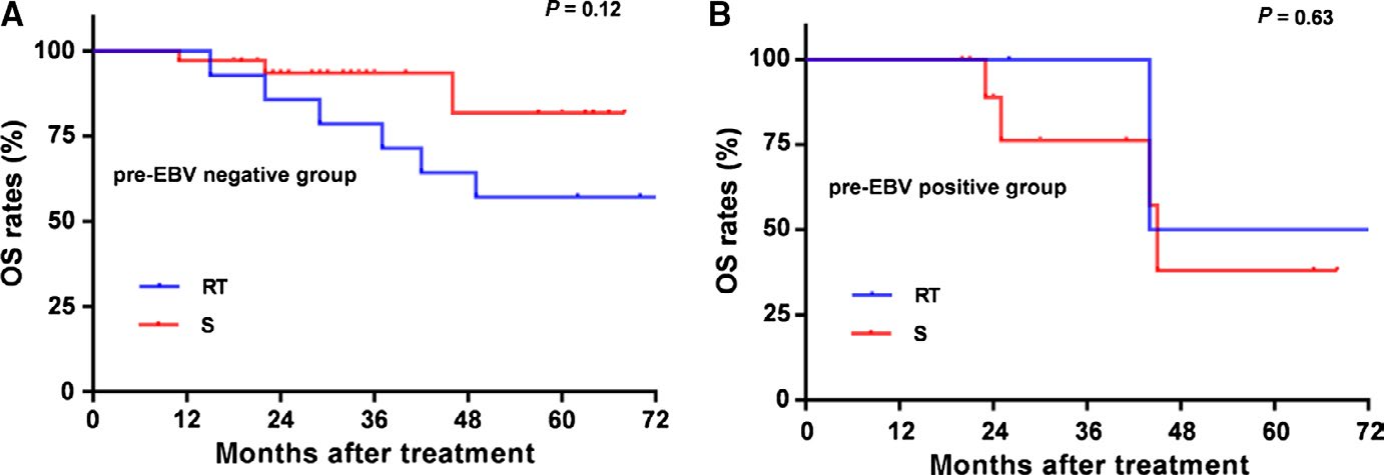
Kaplan‐Meier Effect of treatment regimen on survival outcomes in patients with rI+rII stage and stratified by different pre‐retreatment EBV statuses. Kaplan–Meier overall survival: (A) for patients with undetectable pre‐retreatment EBV and (B) for patients with detectable pre‐retreatment EBV stratified by RT alone or S alone. OS, overall survival; RT, radiotherapy; S, surgery

In this study, there were 66 of 401 patients with an rI + rII stage. Among these, 17 (25.8%) patients accepted RT alone and 49 (74.2%) received S alone. There was no significant difference between RT and S in LRRFS, DMFS, or OS (all *P* > 0.05; Figure 3). Moreover, we stratified these patients according to the pre EBV status. There were 49 patients who were pre EBV‐negative and 17 patients who were pre EBV‐positive. Additionally, there were no significant differences in OS between RT and S in the pre EBV‐negative group or the pre EBV‐positive group (both *P* > 0.05; Figure 4.).

## 4 DISCUSSION

In general, the clinical value of pretreatment EBV DNA in patients with primary NPC has been widely demonstrated. The value of pre‐retreatment plasma EBV DNA in patients with lrNPC has not yet been evaluated before, and this study is the first one to describe the level of pre‐retreatment plasma EBV in patients diagnosed with lrNPC and to assess the prognostic value of pre‐retreatment plasma EBV on survival of patients with lrNPC. A recent study by Chan et al^33^showed that the plasma EBV DNA level was correlated with T category and resection margin status in a cohort of 60 patients with lrNPC received salvage surgery only. The mean concentration of preoperative plasma EBV DNA was associated with tumor load (T1: 48 copies/mL, T2: 316 copies/mL, T3:890 copies/mL, *P* = 0.03). With the above‐mentioned findings, plasma EBV DNA concentration can reflect the tumor load burden in NPC.^10^ In the present study, it was also applied to lrNPC. The mean pre‐retreatment plasma EBV DNA has strong relations with recurrent T category, recurrent N category, and recurrent clinical stage, and the patient with advanced‐stage lrNPC had higher levels of pre‐retreatment plasma EBV DNA (all *P* < 0.001). According to previous studies, pretreatment plasma EBV DNA was detectable (>0 copy/mL) in 53.9%–93% of patients with NPC. In contrast, in this study, pre‐retreatment plasma EBV DNA was detectable (>0 copy/mL) in 49.1% of patients with lrNPC, which was lower than primary NPC. The mechanism is still unknown to us, and an explanation is that locoregionally recurrent cancer cells often regrow from irradiated sites. Post‐irradiation changes such as stromal fibrosis and decreased vascularity may interfere with the release of EBV DNA into the plasma.

Recent studies demonstrated that the presence of severe late complications, rT3‐4 category and tumor volume were independent prognostic factors for OS in patients with rNPC.^34^, ^35^, ^36^ However, in primary NPC, there was a finding that patients with small tumor volume and high EBV DNA level had a worse prognosis than those with large tumor and low EBV DNA level. Patients with low EBV DNA levels, and either small or large tumor volumes, had favorable prognosis.^19^ A recent study showed that high levels of pre EBV with early stage (I + II) disease demonstrated higher OS than low levels of pre EBV with advanced stage (III + IVAB) disease, but not DMFS in newly diagnosed NPC.^37^ Thus, the value of plasma EBV DNA and recurrent overall stage for predicting survival outcomes are still controversial in patients with lrNPC. In this study, subgroup analysis of survival outcomes according to the 8th edition of the AJCC also demonstrated that advanced recurrent T category and recurrent clinical stage were associated with poorer survival (both *P* < 0.001).

A study by An X et al^38^ reported that metastatic/recurrent NPC patients with low pretreatment plasma EBV DNA level and undetectable posttreatment plasma EBV DNA showed a favorable prognosis (5‐year OS was 50.6%), which indicated that plasma EBV DNA had predictive value for prognosis in metastatic/recurrent NPC patients undergoing palliative chemotherapy. Moreover, in our study, the outcomes of univariate analysis may be less convincing on account of the unbalanced distribution of recurrent tumor stage and treatment protocols, while there are no exactly standard therapeutic modalities in patients with lrNPC. With the developed surgical technology, nasopharyngectomy is recommended for early stage resectable disease.^33^, ^35^, ^39^ Comparing the efficacy of surgery with re‐irradiation is difficult as patients eligible for surgery generally have earlier stage disease (usually rT1‐2), smaller tumor volume and fewer comorbidities. There was no randomized controlled study comparing the two modalities, but retrospective case series suggested that their local control rates were probably similar if dealing with the same group of patients.^40^ In our study, subgroup analysis of treatment modalities in patients in rI + rII stage indicated that there was no significant difference in OS between treatment with RT alone or S alone, even when further stratified by pre EBV status (all *P* > 0.05). On the other hand, an explanation is that the proportion of detected pre‐retreatment plasma EBV DNA in lrNPC is lower than the pretreatment EBV DNA in primary NPC, especially in early stage.

Some recent studies showed that ^125^I brachytherapy was a feasible, safe, and effective treatment for locally recurrent NPC,^41^ and the 3D high‐definition endoscopic system improves the precision of endoscopic nasopharyngectomy, particularly when dissection of the internal carotid artery and dura is required.^42^ With advances in technology, the appropriate treatment protocol for patients with detected pre EBV DNA may need further study. Additionally, the results of multivariate analysis showed that patients with undetected pre EBV DNA have an obviously better prognosis than patients with detected pre EBV DNA, and recurrent clinical stage is as ever the most significant prognostic factor for OS. Pre EBV DNA status is an independent prognostic factor for LRRFS, but not DMFS or OS in lrNPC, which is different from primary NPC. Moreover, recurrent clinical stage was also an independent prognostic factor for LRRFS, which is similar to primary NPC. Additionally, early PET‐CT response and plasma EBV DNA clearance could predict survival and subsequent response to chemotherapy in patients with advanced or recurrent NPC.^43^ Here, findings indicate that the high level of pre‐retreatment plasma EBV DNA may imply the possibility for second recurrent failure and poor OS, which hints at more careful examinations, such as a whole‐body scan (eg, PET/CT) and routine follow‐up EBV DNA assays may need to be performed at regular intervals.

Investigation of EBV miRNA target genes revealed the inhibition of tumor suppressor genes and upregulation of multiple EBV‐encoded miRNAs in NPC.^44^ The BART miRNA cluster was related to the expression of LMP1, Zp, gp350, and EBNA1.^45^ Chan et al demonstrated that tissue EBV microRNA BART7 is useful for identifying a subgroup of patients with histologically clear margins who were at increased risk of subsequent local tumor recurrence.^46^ Considering the finding that patients with lrNPC and high EBV DNA levels were at greater risk of second local recurrence. Latent infection is known to be necessary for virus persistence and immune evasion and the EBV after lysis will express a large number of viral proteins to induce immune recognition and attack, so maintaining stable latent infection is an important means of EBV immune escape.^47^ And the challenge in treating lrNPC of the development of an effective EBV vaccine as an immunotherapeutic strategy for primary and recurrent NPC may be important and needed in the future.

Moreover, the present study has several limitations. First, biases due to the retrospective nature of the analyses are unavoidable when the follow‐up time is inadequate and it was a single‐center study. Second, not all of the patients with tumor recurrence underwent histologic examinations or PET/CT scans. Despite these limitations above, the highlights of this study are that it is the first large‐scale study to evaluate the importance of pre‐retreatment EBV DNA and its value for predicting survival. In summary, future prospective, multicenter clinical studies should be warranted to verify the results of this current study.

## 5 CONCLUSIONS

The positive rate of pre‐retreatment plasma EBV DNA in lrNPC is lower than primary NPC. The prognosis of EBV DNA negative group is better than positive group. For locally early‐stage lrNPC, regardless of EBV DNA status, radiotherapy and surgery are available options and both can achieve better long‐term survival.

## Data Availability

The data that support the findings of this study are available on request from the corresponding author. The data are not publicly available due to privacy or ethical restrictions.
